# Hypersexuality Addiction and Withdrawal: Phenomenology, Neurogenetics and Epigenetics

**DOI:** 10.7759/cureus.348

**Published:** 2015-10-12

**Authors:** Kenneth Blum, Rajendra D Badgaiyan, Mark S Gold

**Affiliations:** 1 Department of Psychiatry, McKnight Brain Institute, University of Florida; 2 Department of Psychiatry, and Laboratory of Advanced Radiochemistry, University of Minnesota School of Medicine; 3 Departments of Psychiatry & Behavioral Sciences, Keck School of Medicine of USC, Los Angeles, CA, USA

**Keywords:** neurogenetics, epigenetics, reward deficiency syndrome, compulsivity, hypersexuality disorder

## Abstract

Hypersexuality has been defined as abnormally increased sexual activity. Epidemiological and clinical studies have shown that this non-paraphilic condition consists of "excessive" sexual behaviors and disorders accompanied by personal distress and social and medical morbidity. It is a very controversial and political topic in terms of how best to categorize it as similar or not similar to addictive behaviors including substance abuse. Hypersexual disorder is conceptualized as a non-paraphilic sexual desire disorder with impulsivity. Pathophysiological perspectives include dysregulation of sexual arousal and desire, sexual impulsivity, and sexual compulsivity. The nucleus accumbens, situated within the ventral striatum, mediates the reinforcing effects of drugs of abuse, such as cocaine, alcohol, nicotine, and food as well as music. Indeed, it is believed that this structure mandates behaviors elicited by incentive stimuli. These behaviors include natural rewards like feeding, drinking, sexual behavior, and exploratory locomotion. An essential rule of positive reinforcement is that motor responses will increase in magnitude and vigor if followed by a rewarding event. Here, we are hypothesizing that there is a common mechanism of action (MOA) for the powerful effects drugs, music, food, and sex have on human motivation. The human drive for the three necessary motivational behaviors "hunger, thirst, and sex" may all have common molecular genetic antecedents that, if impaired, lead to aberrant behaviors. We hypothesize that based on a plethora of scientific support hypersexual activity is indeed like drugs, food, and music that activate brain mesolimbic reward circuitry. Moreover, dopaminergic gene and possibly other candidate neurotransmitter-related gene polymorphisms affect both hedonic and anhedonic behavioral outcomes. There is little known about both the genetics and epigenetics of hypersexuality in the current literature. However, we anticipate that future studies based on assessments with clinical instruments combined with genotyping of sex addicts will provide evidence for specific clustering of sexual typologies with polymorphic associations. There have been some studies using electrophysiological techniques that do not support the view that hypersexuality is indeed similar to substance abuse and other behavioral addictions. The authors are also encouraging both clinical and academic scientists to embark on research using neuroimaging tools to examine natural dopaminergic agonistic agents targeting specific gene polymorphisms to "normalize" hypersexual behavior.

## Introduction and background

Certainly, hypersexual behavior has been documented within clinical and research settings over the past decade [1]. Benjamin Rush, a physician one of the founding father of the United States clinically documented excessive sexual behaviors [2] Richard von Krafft-Ebing, a 19th-century Western European pioneer sexologist, and Hirshfeld in 1948 both continued the work [3-4]. The basic tenant of these investigators suggested that hypersexuality constituted persistent socially deviant sexual behavior(s) in both males and females with excessive sexual appetite being maladaptive. In 1975, Stroller characterized the condition as Don Juanism [5]. In 1969, Allen suggested satyriasis for males and nymphomania in females supported by Ellis and Sagarin [6-7]. Even though hypersexuality is not included as  a psychiatric diagnosis in DSM, work of a number of contemporary investigators including Kafka, Reid, Bancroft, their colleagues and the inclination of the World Health organization could lead to inclusion of this condition as a separate diagnostic entity [8-11].

## Review

### Literature methodology

The Medline database, as of July 12, 2015, was used to perform an internet-based literature search. The following terms were included: hypersexual (170), hypersexuality (479), sexual addiction (1,652), sex addict (1,842), sexual impulsivity (989), compulsive sexual (946), compulsive sex (1,512), sexual compulsion (782), paraphilia-related disorder (234), and excessive sexual (857). Since this article is a brief review rather than a meta-analysis, it is based on a representative selection of these studies that have relevance to the subtopics covered. The non-inclusion of any particular study does not negate its importance to the field. Understandably, there are those that do not agree with the concept that sex addiction is indeed a real disorder and may even have evidence to show that they are on solid ground. However, we argue that there is ample evidence to propose that sexual addiction does exist and studies involving neuroimaging, neurogenetics and even epigenetics support the notion that compulsive sex addiction as well as hypersexuality could be considered as an addictive disorder. While we are cognizant of this discrepancy we respectively submit our point of view to generate further scientific scrutiny and not to dispel scientific rhetoric on this important subject. To shed additional light on the subject we have searched the literature for some consensus. On August 17, 2015 we searched PUBMED Central using the following term -- "Is sex a real addiction?" and retrieved 46 articles.

### Definition of sexual addiction

Sexual addiction is defined as any compulsive sexual behavior that interferes with normal living and causes severe stress on the family, friends, loved ones, and one's work environment. Sexual addiction has been called sexual dependency, hypersexuality, and sexual compulsivity. By any name, it is a compulsive behavior that completely dominates the addict's life. Sexual addicts make sex a priority more important than family, friends, and work. Sex becomes the organizing principle of addict's lives. They are willing to sacrifice what they cherish most to preserve and continue their unhealthy behavior [12]. Hypersexual desire has been delineated as desire based on a lifetime assessment of the frequency of sexual behavior and time spent in associated sexual fantasies. In males, an evaluation of hypersexual desire was defined by Kafka and Hannen as the highest sustained period (at least six months minimum duration) of persistently enacted sexual behavior (total sexual outlet/Week after age 15). In fact, a longitudinal history of hypersexual desire, operationally defined as above, was identified in 72-80% of males seeking treatment for paraphilias and paraphilia-related disorders [13].

### Hypersexuality and gender differences

It is well established that in the human sexual community and literature sexual desire is capitulated as the presence of sexual fantasies, activities or urges, and motivation by the human to engage in sexual behaviors. There are both internal and external relevant cues [14]. Evolutionary theory proponents have argued that men and women have different agendas when it comes to sexual activity [15]. Numerous studies reveal distinct differences between males and females. Males have increased sexual fantasy [16], increased frequency of masturbation [17], increased propensity for externally generated visual sexual arousal [18] permissive attitudes toward casual sex [19], ease of arousal [20], and intrinsic motivation [21]. In contrast, females show a different sexual landscape with sexual motivation, sexual arousal, and sexual behavior being shaped by evolutionary factors [22] and greater biological, emotional, and temporal investment in reproduction and child-rearing [23]. Females are less vulnerable to hypersexuality [24] and adapted to foster affiliative relationships and longer-term partner commitment [25]. While sexual addiction is estimated to afflict up to 3% to 6% of the population, the clear understanding of the neurobiological antecedents are limited [26] as well as clinical assessments [27]. We encourage further reading on sexual compulsion, attachment and sexual orientation [28], and gender differences in responses to sexual stimuli [29-30].

It is noteworthy that Kafka and Hennen [13], found that the mean age of onset of persistent hypersexual behavior was 18.7±7.2 years in sexually active men and the age range of onset of hypersexual behavior was age 7-46. The average duration of this highest consistently maintained frequency of sexual appetitive behavior was 12.3±10.1 years. However, the mean age of these active sexual males being hypersexual that sought treatment was 37±9 years. Hanson, et al. also evaluated hypersexuality in offenders and found that low offenders had lower recidivism rates than high-risk offenders [31].

### Hypersexuality and co-morbid substance abuse

There is a high co-morbidity between hypersexual disorder and other addictions, such as substance use disorder [32-33]. Specifically, Garcia and Thibaut proposed that the phenomenology of excessive non-paraphilic sexual disorder should be classified as an addictive behavior, rather than an obsessive-compulsive, or an impulse control disorder [34]. They correctly point out that the criteria are quite close to those of addictive disorders as also proposed by others [35]. These investigators have provided the impetus for continued research in this area and possible future inclusion of hypersexual disorder in the DSM-6. Drugs of abuse, rock ’n' roll, and sex are co-occurring, and entire festivals have been built around these combinations from Woodstock to the present.

The literature reveals that users of methamphetamine report that this stimulatory drug increases sexual desire, especially risky behavior. However, amphetamine has been shown to reduce the sexual activity of female rats. With this in mind, Holder, et al. evaluated the role of methamphetamine in female rats [36]. They found that, on the contrary, methamphetamine facilitated female sexual behavior, and this effect is due to enhancement of dopaminergic transmission and even possible neurotransmission due to the combination of ovarian hormones and methamphetamine. Specifically, they found an enhancement of sexual motivation coupled with activation of neuronal activity in the medial amygdala and ventromedial nucleus of the hypothalamus.

Moreover, scientists from the Netherlands have studied the co-morbidity of substance abuse in self-identified swingers [37]. In this study, Spauwen, et al. concluded that 79% of swingers reported recreational drug use (including alcohol and use of erectile dysfunction drugs); 46% of them reported multiple drug use. In fact, recreational drug use (excluding alcohol and erectile dysfunction drugs) was significantly linked with high-risk sexual behaviors in men and women. Also, drug use was independently associated with sexually transmitted infections (STI) in female swingers, especially those who participate in group sex.

Castelo-Branco, et al. reported that young adult women perceive that sexuality is an important part of their life but not a primary concern (77.6%) [38]. They also reported that alcohol removes the barriers to having sex (62.3%). Importantly, they also found that alcohol abuse was a predictive variable in enhancing risky behaviors independent of the age of the female.

It is noteworthy that Jia, et al. reported dangerous sexual behaviors among psychostimulant and heroin abusers, including multiple sexual intercourse, casual sexual partners, homosexual partners, and never or occasionally practicing safe sex [39].

Our main tenant is that drugs, such as methamphetamine, cocaine, heroin, and alcohol, can stimulate sexual desire in non-addicts. In addicts, it is quite different; the same drugs can cause anhedonia on a chronic basis. However, post-addiction during the recovery phase in many cases aphrodisiac-like behaviors have been observed.

### Hypersexuality and withdrawal

A PubMed search (7-19-15) using the term "hypersexuality and withdrawal symptoms" resulted in only five articles, none of which described "withdrawal symptomatology." However, an alternative search using the terms "high sexual activity withdrawal symptoms" resulted in 25 listed articles.

Addicts in recovery report increases in eating and an appetite drive for certain foods and abuse of cigarettes during prolonged abstinence. Weight gain has also been demonstrated and documented in recently and prolonged abstinent animals and humans [39]. Bruijnzeel made the interesting observation that acute opiate withdrawal can result in spontaneous orgasms [40]. Importantly, in the article, Bruijnzeel proposed that withdrawal symptomatology from drugs and possibly chronic intensive sexual activity may be due to the unopposed function of kappa opioid receptor signaling that inhibits the release of dopamine while increasing norepinephrine in brain reward circuits.

The papers in the literature suffered from a lack of rigor regarding acute and prolonged withdrawal and abstinence confirmed by urine testing. Clinical treatment programs have increasingly moved from treating both genders to isolating the two sexes, providing gender­ specific treatment programs. They also offer education to address the early and prolonged increases in sexual interest and activity, and the relationship of recovery to food and overeating.

Hypersexuality-induced withdrawal symptoms have been reported by some investigators with varying degrees of severity and co-morbid substance abuse [41-45]. As a result of this search, we did not find a single paper describing actual withdrawal symptoms associated with abstinence from active sexual encounters. Most of the papers cited involved the effects of withdrawal from drugs of abuse, such as opioids, nicotine, amphetamines, and cocaine, which can impair sexual activity.

### Hypersexuality and neurogenetics

A PubMed search (7-19-15) revealed only six listed papers using the term "genes and hypersexuality" mostly focusing on articles related to Kleine-Levin syndrome (KLS), a very rare disease whereby hypersexuality could last up to 27 years. In one study, it was found that an immune responsive HLA-DQBl, DQBl *0602 was detected in significant quantities in patients with KLS and could elevate the risk of KLS [46-47].

However, when we used the terms "sexual activity and genes," 2,826 articles were listed, and we provide a brief synopsis of a few important neurogenetic aspects. It is our hypothesis that both hedonic and anhedonic behaviors are outcomes in part of an individual's risk alleles for these behaviors and that treatment consists of appropriately targeting these identified polymorphisms. Moreover, treatment response also depends on these risk alleles and provides an important rationale for pharmacogenetic testing and pharmacogenomic/nutrigenomic solutions.

Following the controversial initial finding by Blum, et al. in 1990 of the first evidence for an association between the DRD2 Al allele and severe alcoholism, there have been 3,938 articles in PubMed (7-19-15) [48]. The studies cover the psychiatric gene polymorphism, the DRD2 Al allele, and associated behaviors and physiology. There is, however, a paucity of data linking sexual activity to this and other related genes despite the overwhelming evidence for mesolimbic activation, especially in dopaminergic pathways and neuronal loci related to sexual stimuli and activity. It is noteworthy that Blum and Noble correctly classified the DRD2 gene as a generalized reward gene responsible for all reward deficiency syndrome (RDS) behavior. In fact, using Bayesian theorem analysis carriers of the Taq Al allele will, over their lifetime, have a 74% chance, that they will rendezvous with one or more reward deficiency syndrome (RDS) behavior [49].

The first association of any gene polymorphism and sexual activity did not occur until 1999 when Miller, et al. evaluated some dopaminergic genes [50]. The basic finding is that the dopaminergic system in the brain seems to play a major role in the regulation of sexual behavior. The relationship between genes for the Dl, D2, and D4 dopamine receptors and age at first sexual intercourse (AFSI) was examined in a sample of 414 non-Hispanic, European-American men and women. A significant association was observed between a DRD2 allele and AFSI and an even stronger association when the DRD2 allele was interacted with a DRDl allele. A constrained regression model was constructed predicting AFSI using sex and a group of nine psychosocial variables as predictors. Adding the DRD2 and the DRD2-by-DRD1 predictors to this model increased the explained variance by 23% and 55%, respectively. The fact that these findings suggest a stronger association among males than among females is in agreement with the recent work of others showing higher sexual stimuli response in males than in females [51]. So maybe "men are from Mars and women from Venus" and this may even be true for cocaine abuse [52].

Specifically, both preclinical and clinical studies have shown sexually dimorphic patterns in behavioral responses to cocaine in all phases of the cocaine addiction process (induction, maintenance, and relapse). Thus, a clear picture is emerging which suggests that there is a biological basis of sex-specific differences in cocaine addiction. These differences result from the disparate regulation of the CNS by male and female gonadal hormones and may be predicted by the presence of DRD2 gene polymorphisms [53]. Moreover, it is known that genetic associations between COMT and various psychiatric phenotypes frequently show differences between men and women. These include the functional Val (158)Met polymorphism in COMT being associated with obsessive-compulsive disorder in men and with anxiety phenotypes in women. Additionally, the Val (158)Met polymorphism in COMT has a greater impact on cognitive function in boys than girls [54].

Miller, et al. did not find an association of the polymorphisms linked to the DRD4 gene and age of first sexual intercourse [50]. However, others found a significant association in certain ethnic groups. Specifically, their analysis of the polymorphisms in DRD4 indicates that those with any - 3R genotype experienced a risk of first sexual intercourse higher than those with other (or any - 4R) genotype in the all-ethnicities (n = 2,552). Interestingly, the risk of first sex does not differ between the two genotypes in the African-American sample, raising the question of cultural upbringing [55].

The sexual experience, like repeated drug use, produces long-term changes, including sensitization in the nucleus accumbens (NAc) and dorsal striatum. Bradley, et al. using microarray analysis to study hamsters found for the first time that the sexual experience in either male or female animals differentially up or down regulates mRNA expression of a series of genes in the NAc [56]. They found that in comparison with sexually naive animals, sexually experienced hamsters receiving a stimulus male on Week 7 exhibited an increase in a large number of genes. Conversely, sexually experienced female hamsters not receiving a stimulus male on Week 7 exhibited a reduction in the expression of many genes. According to the authors, this first ever gene profiling in female hamsters may provide an insight into the mechanisms by which both behaviors (sex) and drugs of abuse induce long-term changes in the mesolimbic and nigrostriatal dopamine pathways.

Bipolar electrodes, implanted bilaterally in the lateral hypothalamus and substantia nigra­ventral tegmental area, stereotaxically were used to provide chronic self-stimulating reward experiences similar to sexual behavior. This type of stimulation has been found to induce a significant increase in the number of synapses in the CA3 region of the hippocampus and the molecular layer of the motor cortex in rats. In essence, chronic brain stimulation induced long­ term potentiation (LTP), which is known to increase new synaptic connections [57]. A single exposure to cocaine in naive animals is sufficient to trigger sustained changes in ventral tegmental area (VTA) glutamatergic synapses that resemble activity-dependent LTP in other brain regions. This cocaine-induced LTP appears to be mediated via dopamine D5 receptor activation of N-methyl-D-aspartate (NMDA) receptors and to require protein synthesis [58], once again supporting our premise proposed here that drugs and sex may have common neurochemical substrates.

Empirical research has revealed a positive relationship between the number of sex partners and involvement in antisocial behaviors [59]. Most attempts to explain this association have taken an evolutionary perspective. From the evolutionary perspective, the same traits, for example, impulsiveness, shortsightedness, and aggressiveness, that are related to a large number of sex partners are also related to criminal involvement. However, there is also reason to believe that the co-variation between sex partners and crime behaviors can be partially explained by a common genetic pathway, where genes that are related to sex partner numbers are also related to antisocial conduct. Using the above-described rationale, Beaver et al. found a strong positive association between sex partners and antisocial behavior and polymorphisms of the dopamine transporter gene (DAT1) explains variation in both numbers of sexual partners and criminal conduct for males [59]. The polymorphic effect of the DAT l gene and the number of sexual partners may be due an association found between certain polymorphisms and male premature penile ejaculation. Carriers of the 1OR/1OR genotype had scores indicating a lower threshold to ejaculate on each of the indicators compared to the combined 9R9R/9R10R (9R higher activity lower dopamine availability) carrier group [60]. Polymorphisms of the DATl gene, specifically the 10R/10R genotype, has been found in juvenile delinquents attending the Brown School (San Marcos, Texas) for pathological aggressive behaviors, including anti-social behavior [61]. A positive correlation of both DRD2 and the DATl polymorphisms were observed with pathological violence in adolescents in a blinded clinical trial. Moreover, though initially conceptualized as resulting from peer imitation of child-onset or life-course-persistent youth, there is mounting evidence from twin studies that adolescent-onset or adolescent-limited antisocial behavior may also be genetically influenced. Burt and Mikolajewski not only confirmed these findings with the DATl gene but extended these findings to include the His452Tyr variant of the gene encoding the 5-HT2A receptor as well [62], More recently, Jozkow et al. reported an association between the sexual dimension of aging males' symptoms (AMS) and genetic variants of 5-HTRlB G861C [63]. Moreover, Sales, et al. found through multivariable logistic regression analysis, an interaction between abuse and the 5-HTTLPR group whereby there was a significant association with non-change status, along with partner communication frequency scores at follow-up [64] Having a history of abuse was significantly associated with greater odds of non-change in condom use post-intervention for only those with the s allele.

It is known that polymorphisms in noncoding regions of the vasopressin la receptor gene (Avpr la) are linked to socio-emotional characteristics in humans, chimpanzees, and voles, and may due to a site-specific variation in gene expression. According to Barrett, et al., the socially monogamous prairie vole offers a unique opportunity to study the neurobiology of monogamy [65]. In fact, vasopressin la receptor (VlaR) signaling is necessary for the formation of the pair bond in males. Interestingly, social prairie voles exhibit greater VlaR binding in the reward processing ventral pallidum than do asocial voles of the same genus. Barrett, et al. found that down-regulation of pallidal VlaR density resulted in a significant impairment in the preference for a mated female partner and a reduction in anxiety-like behavior in adulthood [65]. Other work by Garcia, et al. revealed that individuals with at least one 7-repeat allele (7R+) of the DRD4 report a greater categorical rate of promiscuous sexual behavior, including having ever had a "one-night stand," and report a more than 50% increase in instances of sexual infidelity [66].

Importantly, Daw and Guo reported that individuals carrying the genotypes DRD2*Al/A2, DRD2*A2/A2, DATl *9R/10R, and MAOA*2R/ are associated with higher odds of unprotected sexual intercourse than other genotypes at these loci [67]. The DRD2 associations apply to both men and women, whereas the other links apply to women only. Finally, Emanuele, et al. reported a significant association between the DRD2 TaqI A genotypes and "Eros· (a loving style characterized by a tendency to develop intense emotional experiences based on the physical attraction to the partner), as well as between the C516T 5HT2A polymorphism and "mania" (a possessive and dependent romantic attachment, characterized by self-defeating emotions) [68].

### Epigenetics and sexual activity

A review of the literature reveals that a number of recent articles point out the importance of epigenetic effects on sexual activity. For example, Matsuda reviewed the epigenetic changes of the estrogen receptor a (ERalpha) and influence on sociosexual behavior [69]. In fact, alteration of ER alpha gene activity mediated by epigenetic mechanisms, such as histone modifications and DNA methylation, alters one's sexual behaviors. In terms of homosexuality, Rice, et al. developed a model that may explain the canalization (conversion) homosexuality sexual development [70]. They explain that this model is based on epigenetic marks laid down in response to the XX vs. XY karyotype in embryonic stem cells. Accordingly, these marks boost sensitivity to testosterone in XY fetuses and lower it in XX fetuses, thereby canalizing sexual development. It has been postulated that a subset of these converting epigenetic marks quantitatively may carry over trans-genetically, and could lead to mosaicism for sexual development in opposite-sex offspring­-the homosexual phenotype.

In the socially monogamous prairie vole (Microtus ochrogaster), mating induces enduring pair­ bonds that are initiated by partner preference formation and regulated by a variety of neurotransmitters, including oxytocin, vasopressin, and dopamine. Work by Gundersen [71], and Wang, et al. [72] suggests that histone deacetylase could facilitate partner formation in female prairie voles that may have relevance to humans. Specifically, Wang, et al. found that histone­deacetylase-inhibitors-sodium butyrate and trichostatin A (TSA) enhanced partner preference formation in female prairie voles [72]. This partner preference formation was associated with an upregulation of oxytocin receptor (OTR, oxtr) and vasopressin V la receptor (VlaR, avprla) in the NAc, through an increase in histone acetylation at their respective promoters.

There is interest growing evidence that indicates that females actively engage in polyandry either to avoid genetic incompatibility or to bias paternity in favor of genetically superior males. There is the possibility that selection of superior male fitness may be due epigenetic effects. According to Zeh and Zeh, unlike DNA sequence-based variation, epigenetic variation can be strongly influenced by environmental and stochastic effects experienced during the lifetime of an individual [73]. They suggest that epigenetic variation may be important for the post-copulatory sexual selection and may account for findings linking sperm competitive ability to offspring fitness.

### Genetic and meme evolution: Human procreation

Eysenck proposed a positive correlation between extraversion and intensified sexual behavior and between neuroticism and problems in sexual behavior (anti-social behavior). An earlier study with married people did not show any of these correlations. It was hypothesized that this connection exists only for unmarried persons not engaged in long-lasting relationships because the quality of the relationship determines the sexual interaction. Within a sample of young unmarried men, there was a positive correlation between extraversion and items in which the person described earlier sexual activity with more individuals and in higher frequency. No correlation was found with neuroticism. There were also slight correlations with other personality and social attitude scales. Because of the correlation with an acting-out personality scale, the findings were interpreted from a social-psychological perspective. In today's society, the young male is expected to take the initiative in a sexual interaction that an extraverted young male can realize better than one who is introverted [74]. This perspective is in direct agreement with Richard Brodie's idea about selfish genes of the mind [75]. From the DNA's point of view, of course, anthropologists would agree "we're still here for one reason only; to go forth and multiply." While evolutionary advances are slow, one step every 20 years or so, compared with "meme evolution, an idea mutates in the time it takes to read a sentence." Our brains have nothing to do with genetic evolution except as it is related to smart people having fewer babies. In fact, if there are genes that give people the tendency to take on memes that limit their number of offspring, they will die out in a few generations in favor of genes that give people a tendency to acquire children. Although somewhat controversial, unfortunately, a number of studies suggest Homo sapiens over the last 42,000 years have lowered their IQs due to selective mating [76].

Extraordinarily, it turns out that since extraversion is linked to increased sexual activity especially in males, quantitative geneticist estimates the heritability of the extraverted personality to be around 40-60%. Smillie and associates studied and found that one copy of the DRD2 gene Al allele was associated with significantly higher extraversion [77]. This association raises an interesting question in terms of human procreation. Comings suggested that because of their marked effect on reproductive behavior, learning disorders and other impulsive, compulsive, aggressive, and addictive disorders those carriers of the DRD2 Al have the potential to cause progressive and permanent changes in the frequency of the DRD2Al allele "leading to the genetic meltdown of the species" [78].

In his book, Comings provides evidence that people with addictive-disruptive behaviors have children earlier, and this impacts the selection of addiction genes like the DRD2 Al allele [79]. He suggests that individuals carrying this disruptive risk allele will have children let's say at 20 years of age and individuals without this allele will have children at 25 years. As a result, the mutant gene will reproduce faster, namely, every 20 years while the normal form of the gene will reproduce every 25 years. The ratio of 25/20 is 1.25. Thus, the rate at which a gene that has a 1.25-fold selective advantage will increase in frequency from generation to generation. A difference of five years in the age of mothers or fathers when they have their first children is sufficient to result in a significant and relatively rapid selection for genes carried by group initiating childbearing at an earlier age. Increases in some RDS behaviors have been documented from 1955 to the present. These increases include adolescent behavior syndrome (drugs, sex, teen pregnancies, and delinquent behaviors, smoking), conduct disorder, crime, drug abuse, alcoholism, unprotected sexual behavior, unwed mothers, welfare, school expelled, and school dropouts, as well as a concomitant decrease in IQ [80]. These results are based on the Berkeley Study utilizing longitudinal data from the Child Health and Developmental Studies and the National Longitudinal Surveys of Youth or NLYS [81]. Utilizing this information, Comings predicted that from 1955 to 2015 there will be a doubling of the frequency of, for example, the DRD2 Al allele, therefore increasing the prevalence of RDS behaviors, including precocious sexual intercourse [50]. We encourage a follow-up of this interesting prediction.

In spite of some disagreement, we are proposing hypersexuality disorder as a subtype of RDS sharing characteristics with substance and non-substance addictive behaviors with its clinical expression being partly affected by both genetics and epigenetics. Although untested at this time, we also propose short-term FDA-approved medication-assisted treatments (MAT) favoring blocking dopamine function followed by gentle activation of dopaminergic pathways leading to long-term dopamine homeostasis. The latter could be accomplished by some modalities that may help in recovery.

**Figure 1:**
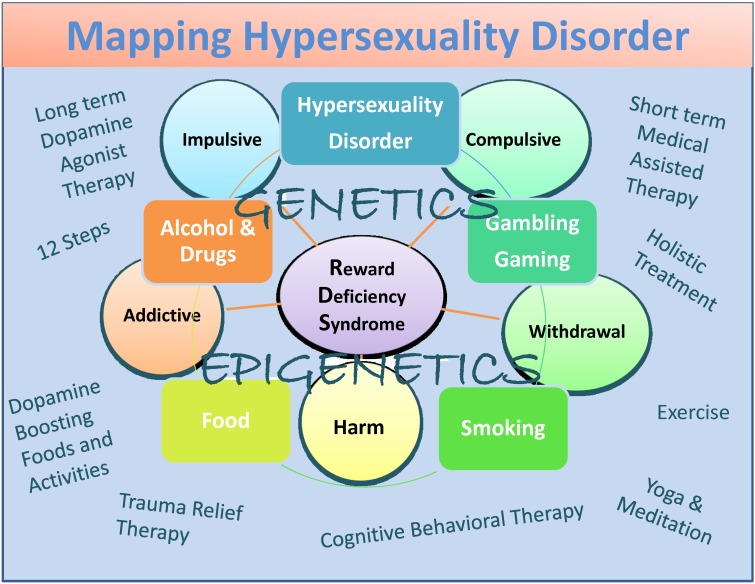
A descriptive map of Hypersexuality Disorder as a subtype of RDS The figure illustrates interactive neurogenetic and epigenetic effects. Both short-term dopamine blocking and long-term "dopaminergic-homeostasis"-based treatments and dopamine boosting therapies and daily activities are listed. Circles indicate RDS characteristics and boxes indicate RDS behaviors.

Albeit potential bias, they include dopamine agonist therapy-nutraceuticals (KB220), the 12 Step program and tradition, holistic treatment, cognitive behavioral therapy (CBT), and trauma relief therapy (TRT) as well as dopamine-boosting activities and foods (Figure 1) [82].

### The controversy

While we firmly believe that hypersexuality disorder should be included in future editions of DSM, we are somewhat perplexed that so little is known about this disorder in terms of neurogenetics and epigenetics and even withdrawal symptomatology and overall phenomenology [83]. The prime take-home message is that we now encourage the scientific community to perform experiments, especially in the realm of neuroimaging and neurogenetics, including epigenetics specific to genes, such as oxytocin-vasopressin-orexin-dopamine as well as other reward genes. Possibly this condition may benefit from treatment that targets reward gene polymorphisms to assist in promoting dopamine homeostasis [84-89]. A number of reviews by Joranby, et al. and Edge and Gold support common treatment opportunities related to shared neurochemical mechanisms in brain reward circuitry as espoused earlier in the RDS concept [90-91].

Historically “sex addiction” was included in DSM - III, however, it was removed from DSM-1V because the consensus of the authors of DSM-1V believed that there was insufficient evidence to merit its conclusion. This decision was fraught with immense emotion by leaders in the field. Following this event, a number of scientists including Kafka, Reid, Prause, and others decided to frame “Hypersexuality” not as a sex addiction but a standalone mental disorder and not as an addiction per se. While their earlier work in 2010 suggested as referenced herein that “hypersexuality” was similar to sex addiction and possibly other addictions, including substance abuse, their more recent work backs off from this contention. Recent work in this area reveals continued controversy. There are a number of electrophysiological-based studies, by Prause’s group that provide some evidence that sexual desire, not hypersexuality, predicts self-regulation of sexual arousal [92]. These investigators suggest in other work that subjects reporting problems regulating their viewing of visual sexual stimuli (VSS) who also reported higher sexual desire showed lower late positive potentials (LPP) in response to VSS. The authors propose that this pattern appears different from substance addiction models [93]. However, while not involving subjects with hypersexual disorder, work by Voon’s group has shown that in compulsive-sexual-behavior subjects, exposed to sexually explicit videos, a greater activity in the neural network similar to that observed in drug-cue-reactivity studies [94]. Greater desire or wanting rather than liking was further associated with activity in this neural network. This work dovetails with theories of incentive motivation [95].

We, the authors of the current article, admit that we have not been privy to all the important interactions that have occurred between the proponents of “Hypersexuality Disorder” and their honest intent to have this disorder included in the current DSM-5. While it failed the so-called “acid-test”, there is every reason to believe that it will be included in future editions of the DSM. It is noteworthy that Steven Hyman, the current NIH director, correctly argued that “the DSM is a poor mirror of clinical and biological realities; a fundamentally new approach to diagnostic classification is needed as researchers uncover novel ways to study and understand mental illness" [96]. Moreover, Casey, et al. proposed that while the DSM considers different disorders as distinct entities, “boundaries between disorders are often not as strict as the DSM suggests” [97].

In 2014, Karila, et al. suggested that sexual addiction, also known as hypersexual disorder, has been ignored by some psychiatrists, in spite of the condition causing serious psychosocial problems. According to these authors, they propose that sexual addiction or hypersexual disorder represents different terms for the same problem. They point out that prevalence rates of sexual addiction-related disorders range from 3% to 6%. Accordingly, the construct of Sexual Addiction/ Hypersexual Disorder displays problematic behaviors including: excessive masturbation, cybersex, pornography use, aberrant sexual behavior with consenting adults, telephone sex, strip club visitation, and other addictive behaviors [98]. Certainly we agree that there may be distinct differences between sexual addiction and hypersexuality as noted by Carvalho, et al. [99], Rettenberger, et al. [100], Kor, et al. [1], Reid, et al. [9], Kafka and Hennen [13], and Prause, et al. [93-94] amongst others.

In summary, we have proposed that, while there are some differences between hypersexuality and sex addiction, more research is required to appropriately categorize these very important conditions. We do agree with the work of Walters, et al. [101] that suggested that individual differences in hypersexuality are quantitative rather than qualitative in nature. They suggest also that hypersexuality is organized along a continuum falling at the upper end of the continuum (Figure 1).

## Conclusions

While recognizing the controversy, we propose that possible differences and similarities between hypersexuality disorder and sex addiction should be adequately investigated using neuroimaging (fMRI, PET, SPECT), optogenetics, candidate and microarray analysis, and epigenetic techniques. We believe that these investigations will provide the basis for inclusion of hypersexuality as a disorder in future editions of the DSM.
